# Supplementary material for: HIVprotI: an integrated web based platform for prediction and design of HIV proteins inhibitors
Source: J Cheminform. 2018 Mar 9;10:12. doi: 10.1186/s13321-018-0266-y (PMC5845081; doi:10.1186/s13321-018-0266-y)
Supplement: Supplementary file 2 — Additional file 2. Source code of HIVProtI web server. [file 13321_2018_266_MOESM2_ESM.zip › HIVprotI_Source-code/hivprotI/weka-3-6-6/doc/weka/gui/Logger.html]

Logger


---


|  |  |  |
| --- | --- | --- |
| **Overview** \| **Package** \| **Class** \| **Tree** \| **Deprecated** \| **Index** \| **Help** \| **Weka's home** | |  |
| **PREV CLASS**   **NEXT CLASS** | **FRAMES**    **NO FRAMES**     **All Classes** |  |
| SUMMARY: NESTED \| FIELD \| CONSTR \| METHOD | DETAIL: FIELD \| CONSTR \| METHOD |  |


---


## weka.gui Interface Logger

**All Known Implementing Classes:**: FlowRunner.SimpleLogger, LogPanel, LogPanel, SysErrLog

---

``` public interface Logger ```

Interface for objects that display log (permanent historical) and
status (transient) messages.

**Version:**
:   $Revision: 1.5 $

**Author:**
:   Len Trigg (trigg@cs.waikato.ac.nz)

---

| **Method Summary** | |
| --- | --- |
| `void` | `logMessage(java.lang.String message)`             Sends the supplied message to the log area. |
| `void` | `statusMessage(java.lang.String message)`             Sends the supplied message to the status line. |

**Method Detail**

### logMessage

```
void logMessage(java.lang.String message)
```

:   Sends the supplied message to the log area. These message will typically
    have the current timestamp prepended, and be viewable as a history.

    :   **Parameters:**: `message` - the log message

---


### statusMessage

```
void statusMessage(java.lang.String message)
```

:   Sends the supplied message to the status line. These messages are
    typically one-line status messages to inform the user of progress
    during processing (i.e. it doesn't matter if the user doesn't happen
    to look at each message)

    :   **Parameters:**: `message` - the status message.


---


|  |  |  |
| --- | --- | --- |
| **Overview** \| **Package** \| **Class** \| **Tree** \| **Deprecated** \| **Index** \| **Help** \| **Weka's home** | |  |
| **PREV CLASS**   **NEXT CLASS** | **FRAMES**    **NO FRAMES**     **All Classes** |  |
| SUMMARY: NESTED \| FIELD \| CONSTR \| METHOD | DETAIL: FIELD \| CONSTR \| METHOD |  |


---
